# *In Vitro* Antimicrobial Activity of Extracts from Plants Used Traditionally in South Africa to Treat Tuberculosis and Related Symptoms

**DOI:** 10.1155/2013/840719

**Published:** 2013-03-05

**Authors:** Balungile Madikizela, Ashwell Rungano Ndhlala, Jeffrey Franklin Finnie, Johannes Van Staden

**Affiliations:** Research Centre for Plant Growth and Development, School of Life Sciences, University of KwaZulu-Natal Pietermaritzburg, Private Bag X01, Scottsville 3209, South Africa

## Abstract

Respiratory ailments are major human killers, especially in developing countries. Tuberculosis (TB) is an infectious disease causing a threat to human healthcare. Many South African plants are used in the traditional treatment of TB and related symptoms, but there has not been a sufficient focus on evaluating their antimicrobial properties. The aim of this study was to evaluate the antimicrobial properties of plants used traditionally to treat TB and related symptoms against microorganisms (*Klebsiella pneumoniae, Staphylococcus aureus,* and *Mycobacterium aurum* A+) associated with respiratory infections using the microdilution assay. Ten plants were selected based on a survey of available literature of medicinal plants used in South Africa for the treatment of TB and related symptoms. The petroleum ether, dichloromethane, 80% ethanol, and water extracts of the selected plants were evaluated for antibacterial activity. Out of 68 extracts tested from different parts of the 10 plant species, 17 showed good antimicrobial activities against at least one or more of the microbial strains tested, with minimum inhibitory concentration ranging from 0.195 to 12.5 mg/mL. The good antimicrobial properties of *Abrus precatorius, Terminalia phanerophlebia, Indigofera arrecta,* and *Pentanisia prunelloides* authenticate their traditional use in the treatment of respiratory diseases. Thus, further pharmacological and phytochemical analysis is required.

## 1. Introduction

A few decades ago, there was enormous optimism about the decline of threatening infectious diseases due to advances in technology and science [1]. However, due to a rise in the transitional nature of infectious diseases, such optimism has not yet been met. Worldwide infectious diseases are still the leading cause of death, especially in developing countries, claiming millions of lives yearly despite the enormous improvements made in human healthcare [2]. Globally, infectious diseases are the second leading cause of death of children and adults under the age of 50, placing severe burdens on the developing world [3, 4]. Within the last few decades, there has been the emergence of about 30 threatening infectious diseases with the majority capable of affecting humans [4]. The treatment of infectious diseases is facing a major problem at present with many microbes developing resistance to widely used antibiotics and antiviral therapies [5–7]. A few examples are the pathogens associated with acquired immunodeficiency syndrome (AIDS) and multidrug-resistant TB (MDRTB). In the coming years, emerging diseases will probably increase due to travel, urbanization, overcrowding, and inadequate healthcare leading to new interactions between human beings and animals as well as other diseases [8]. Thus, addressing the problem of infectious diseases is now an important and urgent requirement.

Respiratory diseases are among the major human killers in the world [9]. TB has been reported to be one of the most serious infectious bacterial diseases, causing a threat to healthcare globally despite the availability of drugs and care centres since the 1940s [7, 9–11]. TB is the most common cause of morbidity and mortality especially when co-infection with HIV-1 occurs and is a pandemic in many parts of the world [12]. TB is the most commonly notified disease in South Africa and the fifth largest cause of death [13, 14]. Approximately 285,000 cases of TB were estimated in South Africa in 2005, and it had the seventh highest population with TB in the world and the second in Africa [14]. 

Due to the important role medicinal plants play in the process of drug discovery and development, they are widely recognised as sources of active antimicrobial metabolites [11]. Several studies have been carried out in South Africa to record the ethnobotanical uses of plants in the treatment of tuberculosis and related symptoms such as coughing, respiratory ailments, and fever [11]. Examples of such detailed studies are recorded in useful reviews of medicinal plants by Watt and Breyer-Brandwijk [36]; Hutchings et al. [15], as well as Van Wyk et al. [17, 29]. Thus, there is a great potential in finding medicinal plants with activity against microorganisms related to respiratory infections.

Most studies done in South Africa on plants used traditionally to treat respiratory ailments have focused on evaluating their antimycobacterial activity against TB causing bacterial strains [13, 14, 37] but with little emphasis on the related symptoms [38, 39]. Therefore, this current study was aimed at evaluating the antimicrobial activity of selected plants against bacteria related to respiratory infection.

## 2. Materials and Methods

### 2.1. Selection and Preparation of Plants for Bioassays

Ten plants were selected based on the available literature of medicinal plants used by various South African tribes in the treatment of TB and related symptoms [11, 15, 17, 20, 29]. The plant materials were collected from the University of KwaZulu-Natal Botanical Garden and Ukulinga Research Farm. Voucher specimens were deposited in the University of KwaZulu-Natal (Pietermaritzburg) Herbarium (NU) for botanical authentication. The species name, family, traditional uses, voucher numbers, parts used, and previously screened activity of the plants used in the study are given in Table 1. The plant materials were oven-dried at 50°C, ground into powders, and stored in airtight containers. Dried plant material (10 g) was extracted sequentially with 200 mL of each of the four solvents, petroleum ether (PE), dichloromethane (DCM), 80% ethanol (EtOH), and water (from polar to nonpolar) by sonication for 1 h at 15°C with temperature being maintained by the addition of ice. The samples were then filtered through Whatman No. 1 filter paper and concentrated to dryness under reduced pressure using a rotary evaporator to obtain crude extracts. The aqueous extracts were dried under reduced pressure by means of a freeze-drier. Crude extracts were then stored in the dark at 10°C until use. The percentage yield for each extract was determined. 

### 2.2. Bacterial Strains and Culture Conditions

Three strains of bacteria were used for antibacterial evaluation against the extracts. The strains were *Klebsiella pneumoniae* (ATCC 13883),* Staphylococcus aureus* (ATCC 12600), which were obtained from the American type culture collection, and *Mycobacterium aurum* A+ from the Microbiology Laboratory, Division of Pharmacology, University of Cape Town. The test organisms were selected because of their respiratory pathogenesis and fast growth rates [14, 40]. The strains were maintained on Mueller-Hinton (MH) agar except for *M*. *aurum *A+ which was maintained on Middlebrook 7H10 agar supplemented with 0.5% glycerol and 10% OADC (oleic acid, albumin, dextrose, and catalase).

### 2.3. Antimicrobial Activity Assays

The minimum inhibitory concentrations (MIC) of plant extracts were determined using the broth microdilution method in 96-well microtitre plates for *S*.* aureus* and *K*. *pneumoniae* as described by Eloff [41] and Jadaun et al. [42] for *M. aurum *A+. The extracts were dissolved in 10% DMSO except for aqueous extracts which were dissolved in sterile distilled water. The test organisms (*S*. *aureus* and *K*. *pneumoniae*) were cultured in MH broth for 24 h, while Middlebrook broth supplemented with 10% OADC and 0.5% glycerol was used to culture *M*. *aurum *A+ for 72 h at 37°C. Neomycin and streptomycin were used as the positive controls whereas broth, 10% DMSO, and water were used as negative controls. For antibacterial testing against *S*. *aureus* and *K*. *pneumonia, *100 *µ*L of water were added in each well and Middlebrook broth supplemented with 0.5% glycerol, and 10% OADC was used for *M*. *aurum *A+. One hundred microliters of solvent controls and test samples were added to the first wells of the microplate starting with a concentration of 100 mg/mL of extracts and 5 mg/mL for the positive control and then two-fold serially diluted down the wells. The optical density of *S*. *aureus* and *K*. *pneumoniae *was adjusted with MH broth to match that of McFarland standard, equivalent to 10^8^ colony forming units at 600 nm, and that of *M*. *aurum* A+ was adjusted to 0.125 at 550 nm using Middlebrook broth enriched with 0.5% glycerol and 10% OADC growth supplement. One hundred microliters of the diluted culture were added to all the wells. The microtitre plates were then incubated for 24 h at 37°C (*S*. *aureus* and *K*. *pneumoniae*) and for 72 h at 37°C (*M*.* aurum* A+). After incubation, 40 *µ*L of *p*-iodonitrotetrazolium violet (INT) indicator (*S*. *aureus* and *K*. *pneumoniae*) and resazurin (*M*. *aurum *A+) were added to evaluate growth inhibition. The results were observed after 30 min for *S*. *aureus* and *K*. *pneumoniae* and 24 h for *M*. *aurum *A+. The lowest concentration containing blue (*M*. *aurum* A+) and clear wells (*S*. *aureus* and *K*. *pneumoniae*) were considered as the MIC values. The assay was repeated twice with two replicates per assay.

## 3. Results and Discussion

The plants selected in this study are all used in South African traditional medicine for the treatment of TB and related symptoms. The percentage yield and MIC values of the plant extract against *K*. *pneumoniae*, *S*. *aureus* and *M*. *aurum *A+ are presented in Table 2. The highest quantity of extract yield was observed from the EtOH extract of *P*. *fruticosa*, whilst the lowest one was from PE extracts of the roots of *T*. *phanerophlebia*. Generally, EtOH was the best extractant giving the highest mass of extracts while PE yielded the lowest mass. The masses of the water extracts were the second highest to EtOH for most of the species extracted. In this study, only MIC values equal to or less than 1.0 mg/mL for crude extracts were considered as exhibiting good activity [43]. Out of 68 extracts tested from different parts of 10 species, 17 were found to have good antimicrobial activities at least against one or more of the strains tested. Twelve extracts showed good activity against *M*. *aurum *A+ and 11 against *S*. *aureus*. It was observed that *M*. *aurum *A+ was the most susceptible bacterium while *K*. *pneumoniae* was the most resistant with only 6 extracts showing good activity. Due to lipopolysaccharides present on their outer membrane, Gram-negative bacteria are usually impermeable to most antibacterial compounds [44, 45]. This can explain the low number of active extracts against *K*. *pneumoniae*.

The water extract of *T*. *phanerophlebia* (leaf) showed the best activity with the lowest MIC value of 0.098 mg/mL among the extracts tested. For water extracts only *T*. *phanerophlebia* leaves exhibited good activity against all bacterial species tested with MIC values ranging from 0.098 to 0.39 mg/mL. The water extracts of *I*. *arrecta* (leaf), *P*. *prunelloides *(leaf), and *T*. *phanerophlebia* (root) also showed good activity against at least one bacterial strain with MIC values ranging from 0.39 to 0.78 mg/mL. This was encouraging as many reports often state that water extracts lack bioactivity [46]. The evaluation of aqueous extracts aims to mimic the traditional use; therefore, the observed antibacterial activities exhibited by water extracts could be of interest. Such plants are candidates for further studies such as antimycobacterial evaluation against TB causing bacterial strains.

Comparing the root and leaf extracts of *I*. *arrecta*, the EtOH extracts of the leaf showed good antibacterial activity against all bacterial species tested with MIC values ranging from 0.39 to 0.78 mg/mL. For *P*. *prunelloides* leaves and roots, the EtOH extracts showed good activity with MIC values ranging from 0.195 to 0.78 mg/mL against almost all bacterial species tested. This is noteworthy as it indicates the presence of bioactive compounds in these plants that might help to isolate drugs that cure ailments related to respiratory infection. The palmitic acid previously isolated from roots of *P*. *prunelloides *by Yff et al. [34] could be responsible for the antimicrobial activity of this plant observed in this study. Yff et al. [34] also did an antiviral test on the root decoction of *P*. *prunelloides* and reported the inhibition of *Influenza A virus*. These results make this plant a potentially effective remedy against respiratory diseases, and the evaluation of palmitic acid against respiratory microbes is required.

For *T*. *phanerophlebia* leaf, root, and twig extracts, the EtOH extracts showed good activity against almost all bacterial strains tested with MIC values ranging from 0.098 to 0.39 mg/mL. This, however, was excluding the leaf extract that did not exhibit good activity against one strain and root extract that also did not show good activity against two strains. In a study on antimicrobial properties done by Shai et al. [27] against *S*. *aureus, Escherichia coli, Enterococcus faecalis,* and *Pseudomonas aeruginosa*, the leaf extracts of *T*. *phanerophlebia* exhibited good antimicrobial activity which was also the case in our study against the tested bacteria. In addition, in a literature investigation conducted by Nair et al. [47], several alkaloids were reported to have been isolated from the stem of *T*. *phanerophlebia *which exhibited cyclooxygenase-2 enzyme activity. The antimicrobial activity observed from different parts of this plant might be due to those alkaloids (cholestane triterpenoids *β*-sitostenone, stigmast-4-ene-3,6-dione, and *β*-sitosterol). It is, therefore, important to evaluate these compounds in bioassays against these microorganisms. Among the bark and root extracts of* F*.* sur,* only the EtOH extract (root) showed good activity with an MIC value of 0.78 mg/mL against *K*.* pneumoniae* and 0.195 mg/mL against *S*. *aureus*. The activity of this plant could be due to the triterpene compounds found in the latex isolated in a previous study by Feleke and Brehane [48], which also warrants testing in similar bioassays.

Among the leaf and stem of *L*.* intermedia*, the EtOH extract (leaf) showed good activity with MIC value of 0.195 against *M*. *aurum *A+ and 0.78 against *S*.* aureus*. These findings are very interesting as they indicate the antimicrobial activity of this plant. Of all the extracts of *A*. *africanus* and *A*. *falcatus, *good activity was observed only in the EtOH extracts with both having an MIC value of 0.39 mg/mL against *M*. *aurum* A+. *Asparagus *species are known to have steroidal saponins as their major bioactive constituents; therefore, the antimycobacterial activity of these two species of *Asparagus* could be due to saponins [49]. However, among all extracts of the leaves and seeds of *A*. *precatorius* subsp. *africanus*, only the EtOH (leaves and seeds) and DCM extracts (leaves) showed good activity with MIC values ranging from 0.195 to 0.78 mg/mL against *M*. *aurum* A+ only. Phytochemical research done in a previous study by Taur and Patil [50] from the root and aerial part of this plant showed the presence of triterpenoids and saponins. Saponins are known to have broad spectrum of pharmacological activities, including antimicrobial properties [51]. In view of the fact that these plants were selected based on their traditional uses against TB and related symptoms, these findings are noteworthy. However, all the extracts of *B*. *grandiflora* and* P*. *fruticosa* did not show good activity against all bacterial species tested despite being reported to be used in the treatment of TB and related symptoms. However, bioactivity cannot be completely ruled out from such plant species as they could be active against other bacterial strains that cause respiratory ailments. The other possible explanation could be that antimicrobial effects of these plants are not mediated through direct inhibition on microbial growth but rather through immunostimulation or the compounds potentially active require metabolic activation by certain enzyme *in vivo *[52]. 

## 4. Conclusion

Two or more extracts of *A*. *precatorius *subsp. *africanus*, *T*. *phanerophlebia*, *I*. *arrecta,* and *P*. *prunelloides *showed good antimicrobial activity against at least one or more of the bacterial strains tested. Therefore, the good antimicrobial properties of *A*. *precatorius *subsp. *africanus*, *T*. *phanerophlebia*, *I*. *arrecta,* and *P*. *prunelloides *found in this study form a good basis for further pharmacological (such as antimycobacterial evaluation against infectious TB microorganisms) and phytochemical investigation, and they validate the traditional use of these plants in the treatment of respiratory diseases in South Africa. The fact that some species of plants did not display good antimicrobial activity does not mean that they may have the same effect *in vivo; *thus, it should be noted that they only demonstrated weak activity *in vitro*. Additionally, weak activity might mean that the plant species are used to treat the symptoms of various respiratory ailments rather than the disease itself.

## Figures and Tables

**Table 1:** Medicinal plants used traditionally in South Africa to treat TB and related symptoms.

Family, species	Voucher specimen number	Traditional use	Parts used	Previously screened activity	References
Amaryllidaceae *Brunsvigia grandiflora* Lindl.	BALUNGI 31	Coughs and colds	Bulbs	Mutagenic and antimutagenic activities of bulbs	[15, 16]
Asparagaceae *Asparagus africanus* Lam.	BALUNGI 36	Tuberculosis	Shoots	Analgesic and anti-inflammatory activities of roots. Two antiprotozoal compounds were isolated	[15, 17–19]
Asparagaceae A*sparagus falcatus* (L.) Oberm.	BALUNGI 44	Tuberculosis	Leaves, roots	Caryophyllene-type sesquiterpene lactone isolated from the leaves was found to have remarkable effect on the proliferation (IC_50_ 1.82 uM). Antibacterial activity of the roots	[20–22]
Fabaceae *Abrus precatorius* subsp. *africanus* Verdc.	BALUNGI 43	Tuberculosis, bronchitis, whooping cough, chest complaints, and asthma	Leaves, roots	Sedative effects of roots. Antibacterial and cytotoxicity activity of seeds. Five compounds were isolated and identified as isoflavan, quinones, and hydroquinones	[15, 23–26]
Combretaceae *Terminalia phanerophlebia* Engl. & Diels	BALUNGI 37	Tuberculosis	Roots	Antibacterial and antifungal activities of the leaves	[20, 27]
Leguminosae *Indigofera arrecta *Benth. ex Harv. & Sond.	BALUNGI 41	Tuberculosis	Root	Antibacterial activity of the leaves	[20, 28]
Moraceae *Ficus sur* Forssk.	BALUNGI 39	Tuberculosis/ulceration of the lung	Bark, root	Antimalarial and antibacterial activities of the leaves.	[15, 29–31]
Polygalaceae *Polygala fruticosa *P. J. Bergius	BALUNGI 35	Tuberculosis, blood purification, intestinal sores,sinusitis, and gonorrhoea	Whole plant	Antibacterial activity against *Gardnerella vaginalis* and toxicity	[11, 29, 32, 33]
Rubiaceae *Pentanisia * *prunelloides * Schinz	BALUNGI 40 and 42	Tuberculosis, chest pain, fever, and toothache	Root	Antibacterial activity against *B*.* subtilis*, *S*. *aureus*, *E*.* coli*,and *K*.* pneumonia*. Antiviral activity of leaves and roots	[15, 29, 34]
Lamiaceae *Leonotis intermedia* Lindl.	BALUNGI 38	Colds, coughs, bronchitis, asthma, tuberculosis, high blood pressure, and jaundice	Leaves, stem	Anti-inflammatory activity	[11, 35]

**Table 2:** The antibacterial (MIC values) effects of plants used traditionally as remedies for the treatment of tuberculosis and related symptoms in South Africa.

Plant species	Plant part	Extract	Extract yield % (DW)	Antibacterial activity MIC (mg/mL)		
				*Klebsiella pneumoniae *	*Mycobacterium aurum* A+	*Staphylococcus aureus *
*A. precatorius* subsp. *africanus *	L	PE	1.03	12.50	12.50	12.50
		DCM	1.24	12.50	**0.78**	12.50
		80% EtOH	3.54	3.13	**0.195**	1.56
		Water	2.66	6.25	12.50	3.13
	S	PE	2.28	12.50	12.50	6.25
		DCM	2.87	12.50	6.25	3.13
		80% EtOH	3.42	6.25	**0.78**	1.56
		Water	2.43	1.56	1.56	3.13

*A*.* africanus *	L	PE	0.40	12.50	6.25	12.50
		DCM	0.50	12.50	6.25	12.50
		80% EtOH	1.50	6.25	**0.39**	6.25
		Water	6.80	1.56	6.25	12.50

*A. falcatus *	L	PE	0.80	12.50	3.13	12.50
		DCM	0.62	12.50	1.56	12.50
		80% EtOH	2.76	6.25	**0.39**	3.13
		Water	1.00	3.13	2.56	1.56

*B. grandiflora *	Bb	PE	2.83	12.50	12.50	12.50
		DCM	0.98	12.50	3.13	6.25
		80% EtOH	4.47	3.13	3.13	6.25
		Water	3.42	3.13	3.13	6.25

*F. sur *	B	PE	0.50	12.50	6.25	12.50
		DCM	0.50	12.50	6.35	12.50
		80% EtOH	0.60	6.25	3.13	6.25
		Water	0.40	6.25	6.25	12.50
	R	PE	3.20	6.25	12.5	12.50
		DCM	0.70	6.25	6.25	12.50
		80% EtOH	7.70	**0.78**	3.13	**0.195**
		Water	2.10	12.50	3.13	3.13

*I. arrecta *	L	PE	2.66	6.25	12.50	12.50
		DCM	1.79	12.50	6.25	12.50
		80% EtOH	11.15	**0.78**	**0.39**	**0.39**
		Water	9.69	12.50	**0.78**	6.25
	R	PE	0.60	12.50	6.25	12.50
		DCM	0.70	12.50	1.56	12.50
		80% EtOH	11.40	3.13	1.56	1.56
		Water	0.80	6.35	12.50	12.50

*L. intermedia *	L	PE	1.02	12.50	6.25	12.50
		DCM	1.00	12.50	12.50	12.50
		80% EtOH	5.62	6.25	**0.195**	**0.78**
		Water	3.20	3.13	3.13	1.56
	St	PE	0.50	6.25	3.13	12.50
		DCM	0.60	6.25	12.50	12.50
		80% EtOH	4.80	3.13	1.56	1.56
		Water	3.00	3.13	1.56	6.25

*P. prunelloides *	L	PE	1.30	6.25	6.25	12.50
		DCM	1.40	12.50	12.50	6.25
		80% EtOH	4.40	3.13	**0.39**	**0.195**
		Water	1.50	3.13	1.56	**0.39**
	R	PE	0.20	6.25	3.13	12.50
		DCM	0.20	6.25	12.50	12.50
		80% EtOH	11.40	**0.39**	**0.78**	**0.78**
		Water	6.76	1.56	1.56	1.56

*P. fruticosa *	Wp	PE	0.73	6.25	1.56	6.25
		DCM	1.04	6.25	1.56	6.25
		80% EtOH	16.67	3.13	3.13	1.56
		Water	4.84	3.13	3.13	6.25

*T. phanerophlebia *	L	PE	0.50	6.25	6.25	6.25
		DCM	1.20	6.25	6.25	6.25
		80% EtOH	16.00	**0.195**	1.56	**0.195**
		Water	9.80	**0.39**	**0.39**	**0.098**
	R	PE	0.10	12.50	6.25	12.50
		DCM	0.30	6.25	3.13	6.25
		80% EtOH	12.90	1.56	3.13	**0.195**
		Water	1.70	3.13	6.25	**0.39**
	T	PE	1.00	6.25	3.13	6.25
		DCM	0.40	6.25	1.56	3.13
		80% EtOH	11.00	**0.195**	**0.195**	**0.39**
		Water	3.70	1.56	1.56	1.56

Neomycin				0.0975		0.0975
Streptomycin					0.195	

L: leaves, R: roots, B: bark, Bb: bulb, S: seeds, St: stem, Wp: whole plant, T: twigs, MIC: minimum inhibitory concentration, DCM: dichloromethane, PE: petroleum ether, EtOH: ethanol, % DW: percentage dry weight Values boldly written are considered very active.
